# Comparative Evaluation of Activated and Nonactivated Microbubbles on the Efficacy of Bleaching Agents

**DOI:** 10.7759/cureus.64040

**Published:** 2024-07-07

**Authors:** Poojitha Suganthakumar, Sihivahanan Dhanasekaran, Vijay Venkatesh

**Affiliations:** 1 Conservative Dentistry and Endodontics, SRM Kattankulathur Dental College and Hospital, SRM Institute of Science and Technology (SRMIST), Chengalpattu, IND

**Keywords:** dental bleaching, ultrasonic activation, bleaching agents, hydrogen peroxide (h2o2), microbubbles`

## Abstract

Introduction: Intracoronal bleaching serves as a conservative option for nonvital teeth that exhibit discoloration. Hydrogen peroxide (H_2_O_2_) is frequently utilized in bleaching processes owing to its capability to produce free radicals. The main drawbacks of the currently available bleaching agents are the occurrence of cervical resorption and the multiple dental visits to achieve the desired result. Therefore, in our study, to address the limitations associated with cervical resorption and extended treatment duration for badly stained teeth, an attempt was made to incorporate a whitening agent (35% H_2_O_2_) with microbubbles.

Aim: This study aimed to compare and evaluate the effect of activated and nonactivated microbubbles on the efficacy of bleaching agents.

Methodology: Forty-five human central incisors were collected and divided into three groups: Group I (HP), H_2_O_2_ plain (*n *= 15) (Control); Group II (HPM), H_2_O_2_-infused microbubbles without ultrasonic activation (*n *= 15) (experimental group); and Group III (HPMU), H_2_O_2_-infused microbubbles with ultrasonic activation (*n *= 15) (experimental group). The crowns were artificially stained. Microbubbles containing 35% H_2_O_2 _were generated using the probe sonication method. The bleaching agent H_2_O_2_ plain (0.04 mL) was syringed into the pulp chamber in group I, while H_2_O_2_-infused microbubbles (0.04 mL) were syringed into group II and group III. Group III was further activated ultrasonically. The evaluation of color shade differences was conducted using the Vita Lumin shade guide at three time points: baseline, day 7, and day 14.

Results: Data regarding color change using Vita shade were investigated for normality using the Kolmogorov Smirnov test and assessed a non-normal distribution. Intergroup comparisons at each particular time interval (baseline, day 7, and day 14) were analyzed using the Kruskal-Wallis H test followed by multiple pairwise comparisons using the Adjusted Bonferroni post hoc test. Intragroup comparisons between different time intervals were analyzed using related samples from Friedman’s test followed by multiple pairwise comparisons using the post hoc Dunn test. The level of statistical significance was determined at *P *< 0.05. There was no statistical difference in the baseline values of all three groups. Group I (HP) exhibited an average increase of three Vita Lumin shade tabs on day 7 and day 14, respectively, whereas Group II (HPM) exhibited an average increase of six and four Vita Lumin shade tabs on day 7 and day 14, respectively, and Group III (HPMU) exhibited an average increase of 10 and 3 Vita Lumin shade tabs on day 7 and day 14, respectively.

Conclusions: Microbubbles containing H_2_O_2_ were more efficient and faster than plain H_2_O_2_ for bleaching, and the efficacy of bleaching was enhanced when activated using ultrasonic technology.

## Introduction

Coronal discoloration is a common occurrence in teeth that have undergone root canal therapy, necessitating aesthetic intervention. Various factors can contribute to this discoloration, including pulpal necrosis, intrapulpal bleeding, remnants of pulp tissue, or the use of endodontic materials [1]. The discoloration of the root-filled teeth can be conservatively managed by employing the walking bleach technique thereby, preserving the natural tooth structure while achieving satisfactory aesthetic outcomes over time [2]. To implement the walking bleach technique, a bleaching agent is sealed within the pulp chamber of the affected tooth. This process is repeated periodically until the desired whitening result is obtained [3]

Hydrogen peroxide (H_2_O_2_) is frequently utilized in bleaching processes owing to its capability to produce free radicals, notably hydroperoxyl, and hydroxyl radicals [4]. These free radicals' capacity to readily penetrate dentinal tubules is ascribed to their low molecular weight. Once inside these tubules, they interfere with the lengthy aromatic chains of pigments responsible for tooth discoloration, leading to the creation of shorter linear chains and consequently lightening the appearance of the tooth [5].

H_2_O_2_-based intracoronal bleaching works specifically well for dentin stains, and pulpal discoloration, and ensures both functional integrity and cosmetic appeal while providing a workable option for improving the appearance of discolored nonvital teeth. Despite its evident advantages, intracoronal bleaching presents challenges such as cervical resorption and the extended duration needed to achieve the desired shade [6]. This prolonged process is attributed to the gradual release of hydroxyl radicals, demanding a higher frequency of appointments.

Microbubbles are geometrically defined, with a gas-filled core situated at their center and a shell composed of biocompatible constituents, including proteins or lipids. These microbubbles, with diameters ranging from 0.1 to 10 µm, experience contraction and expansion when exposed to ultrasound. The dynamic nature of this phenomenon leads to the reflection of ultrasonic radiation, the formation of cavitation, and the possibility of microbubble shell rupture [7].

A literature search did not reveal any exploration of the application of ultrasonic activation on microbubbles that have been combined with bleaching chemicals. Thus, the objective of this research is to assess the efficacy of utilizing ultrasonic activation during ex vivo intracoronal bleaching of bleaching chemicals that are integrated within microbubbles thereby augmenting and simultaneously maintaining the liberation of free radicals and facilitating expedited outcomes while minimizing any potential adverse consequences.

The null hypothesis suggests that the introduction of a bleaching agent into microbubbles, with ultrasonic activation, does not enhance the penetration of bleaching agent molecules into dentinal tubules, consequently leading to reduced bleaching effectiveness.

## Materials and methods

The sample size was estimated using the G*Power 3.1 software (Heinrich-Heine-Universität Düsseldorf, Düsseldorf, Germany). Accordingly, for the analysis with an alpha-type error of 0.05 and 95% power, it was determined that the sample size should be 15 for each group.

A total of 45 human upper incisors were extracted as a result of periodontal issues. Patients were informed and gave their consent regarding the use of their extracted teeth for research purposes before the tooth extraction procedure. All upper incisors that were unaffected by cavities, cracks, restorations, endodontic therapy, vertical fractures, or resorptions were included.

Forty-five extracted human central incisors were divided into three groups (Table 1).

**Table 1:** Groups and sample size.

Group	Bleaching agent	Sample size
Group I	Hydrogen peroxide plain (HP)	Control group (*n* = 15)
Group II	Hydrogen peroxide-infused microbubbles without ultrasonic activation (HPM)	Experimental group (*n* = 15)
Group III	Hydrogen peroxide-infused microbubbles with ultrasonic activation (HPMU)	Experimental group (*n* = 15)

Following the extraction, a gauze soaked in a solution of 2.5% sodium hypochlorite was utilized to eliminate any soft tissue that was covering the surface of the root. Additionally, any calculus present was eliminated using scalers. Subsequently, the teeth were preserved in thymol saline solution before being artificially stained with whole blood [5].

The crowns were artificially stained using a modified process derived from the research conducted by Freccia and Peters [8]. The teeth were rinsed with running water, and the cavities were flushed with deionized water after the staining procedure. A procedure involving a gutta-percha cone and AH Plus sealer was employed to fill the canals with gutta-percha. Following 24 hours, the gutta-percha matrix was removed to a depth of 3 mm below the cementoenamel junction, and finally, zinc phosphate cement was used as a cervical plug [5,9].

Numerous processes are employed in the fabrication of microbubbles, such as high shear emulsification, coacervation or coalescence thin-layer evaporation, mechanical agitation, and sonication. In this study, a probe sonicator was employed to generate microbubbles containing 35% H_2_O_2_. Polylactic acid (PLC) is employed as a polymer to generate microbubbles. The microbubbles along with the bleaching agent are dissolved in organic solvents before probe sonication. They are then centrifuged, lyophilized, and filled with a suitable gas, such as CO_2_, to obtain microbubbles loaded with hydrogen peroxide [10].

The bleaching agent H_2_O_2_ plain (HP, 0.04 mL) was syringed into the pulp chamber in Group I, while H_2_O_2_-infused microbubbles (0.04 mL) were syringed into Group II and Group III. The pulp chamber was then gently filled with a plugger or paper points. The bleaching agent in Group III was subjected to ultrasonic activation for 30 seconds without any subsequent cooling. A minimum thickness of 3-4 mm, such as cavit or IRM was used as a temporary seal. The evaluation of color shade differences was conducted using the Vita Lumin shade guide (VITA Zahnfabrik, Bad Säckingen, Germany) under standardized lighting conditions at three-time points: baseline, day 7, and day 14. The data were gathered, organized, and analyzed using statistical methods [11].

Statistical analysis

Data regarding color change using Vita Tab were entered into Microsoft Excel and analyzed using IBM SPSS Statistics for Windows, Version 20 (IBM Corp., Armonk, NY). Data were investigated for normality using the Kolmogorov-Smirnov test, indicating a non-normal distribution. Descriptive statistics were derived as mean, standard deviation, median, and 95% confidence interval. Intergroup comparisons at each particular time interval (baseline, day 7, and day 14) were analyzed using the Kruskal-Wallis H test followed by multiple pairwise comparisons using the adjusted Bonferonni post hoc test. Intragroup comparisons between different time intervals were analyzed using related samples from Friedman’s test followed by multiple pairwise comparisons using the post hoc Dunn test. The level of statistical significance was determined at *P *≤ 0.05.

## Results

**Figure 1:**
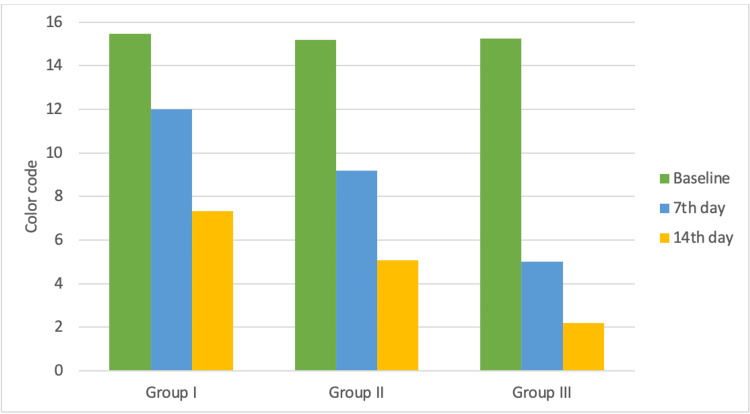
Mean color change in control and experimental groups at baseline, day 7, and day 14.

The findings of this study demonstrate a statistically significant relationship between group I (HP), group II (HPM), and group III (HPMU) on both day 7 and day 14. There was no statistical difference in the baseline values of all three groups. Group I (HP) exhibited an average increase of three Vita Lumin shade tabs on day 7 and day 14, respectively, whereas group II (HPM) exhibited an average increase of six and four Vita Lumin shade tabs on day 7 and day 14, respectively, and group III (HPMU) exhibited an average increase of 10 and 3 Vita Lumin shade tabs on day 7 and day 14, respectively (Tables 2-3 and Figure 1).

**Table 2:** Intergroup comparisons at baseline, day 7, and day 14. ^*^Statistically significant (*P *≤ 0.05). ^†^Adjusted Bonferonni test. Group I,  Control; Group II, hydrogen peroxide without ultrasonic activation; Group III, hydrogen peroxide with ultrasonic activation

Time	Groups	n	Mean ± SD	Median	Min.-Max.	Mean rank	Kruskal-Wallis test value (*P*-value)	Post hoc analysis^†^		
								Comparisons	Test value	*P*-value
Baseline	I	15	15.46 ± 0.74	16	14-16	25.33	0.879 (0.664)	I vs. II	-	-
	II	15	15.20 ± 0.86	15	14-16	21.47		I vs. III	-	-
	III	15	15.26 ± 0.79	15	14-16	22.20		II vs. III	-	-
Day 7	I	15	12.00 ± 0.75	12	11-13	38.00	39.804 (0.000)*	I vs. II	15.000	0.005*
	II	15	9.20 ± 0.67	9	8-10	23.00		I vs. III	30.000	0.000*
	III	15	5.00 ± 0.75	5	4-6	8.00		II vs. III	15.000	0.005*
Day 14	I	15	7.33 ± 1.04	7	6-9	37.37	38.403 (0.000)*	I vs. II	13.733	0.011*
	II	15	5.06 ± 0.79	5	4-7	23.63		I vs. III	29.367	0.000*
	III	15	2.20 ± 0.67	2	1-3	8.00		II vs. III	15.633	0.003*

**Table 3:** Intragroup comparisons at specific time intervals. *Statistically significant (*P *≤ 0.05). ^†^Dunn test. Group I, Control; Group II, hydrogen peroxide without ultrasonic activation; Group III, hydrogen peroxide with ultrasonic activation

Groups	Time intervals	Mean ± SD	Mean rank	Friedman test value (*P*-value)	Post hoc analysis^†^		
					Comparisons	Test value	*P*-value
I	Baseline	15.46 ± 0.74	3.00	30.000 (0.000)*	Baseline vs. day 7	1.000	0.019*
	Day 7	12.00 ± 0.75	2.00		Baseline vs. day 14	2.000	0.000*
	Day 14	7.33 ± 1.04	1.00		Day 7 vs. day 14	1.000	0.019*
II	Baseline	15.20 ± 0.86	3.00	30.000 (0.000)*	Baseline vs. day 7	1.000	0.019*
	Day 7	9.20 ± 0.67	2.00		Baseline vs. day 14	2.000	0.000*
	Day 14	5.06 ± 0.79	1.00		Day 7 vs. day 14	1.000	0.019*
III	Baseline	15.26 ± 0.79	3.00	30.000 (0.000)*	Baseline vs. day 7	1.000	0.019*
	Day 7	5.00 ± 0.75	2.00		Baseline vs. day 14	2.000	0.000*
	Day 14	2.20 ± 0.67	1.00		Day 7 vs. day 14	1.000	0.019*

## Discussion

Intracoronal bleaching is a conservative option for more intricate and intrusive cosmetic interventions for nonvital teeth that exhibit discoloration [12]. The main indication for internal bleaching is intrinsic (internal) discoloration. These discolorations can have various causes, either local or systemic, and are different from those that cause extrinsic (external) staining [13].

The earliest mention of bleaching nonvital teeth was made by Garretson in 1895, who used chlorine as the bleaching chemical [14]. Nevertheless, the utilization of H_2_O_2_ to whiten nonvital teeth did not occur until the year 1951, as documented by Pearson in 1951 [15]. The H_2_O_2_ solutions and gels often employed in dentistry for dental whitening typically have concentrations ranging from 1.5% to 35% [16]. H_2_O_2_ due to its low molecular weight, can infiltrate the dentin and liberate oxygen, disrupting the bivalent bonds of organic and inorganic substances within the dentinal tubule [17].

The findings indicate that the bleaching agent's most significant impact would have ended at the 75-hour mark, with the highest bleaching activity achieved at 27 hours [18]. The main drawbacks of the currently available bleaching agents are the occurrence of cervical resorption and the multiple dental visits to achieve the desired result. The growing need for teeth whitening has prompted numerous manufacturers and researchers to enhance the development of bleaching products that yield expedited and safer outcomes.

Several studies have discovered that the penetration of H_2_O_2_ is improved by higher concentrations of H_2_O_2_, longer application times, elevated temperature, larger openings in the dentinal tubules of young teeth, variations in the position of the tooth's structure, acid etching, and light activation. In addition, the use of particular formulations and delivery technologies has further enhanced the ability of substances to penetrate [19]. Therefore, in our study, to address the limitations associated with cervical resorption and extended treatment duration for badly stained teeth, an attempt was made to incorporate a whitening agent (35% H_2_O_2_) with microbubbles.

Microbubbles are gas-carrying concavities present in aqueous solutions, characterized by their size range being less than 1 μm. The bubbles exhibit a spherical shape and possess a shell composed of lipids, proteins, polymers, surfactants, polyelectrolyte multilayers, and a core filled with gas, which allows them to exhibit observable dynamic characteristics [8]. Upon exposure to ultrasonic waves, the microbubbles have the potential to undergo oscillation and subsequent collapse. The release of the bleaching chemical into dentinal tubules is facilitated by these microbubbles. Consequently, the concentration of H_2_O_2 _exhibited a significant increase after ultrasonic treatment. In the current investigation, the ultrasonic apparatus was engaged at a minimum energy level of 24 kHz for 30 seconds to prevent excessive temperature elevation within the pulp chamber [5].

Visual color determination is well recognized as subjective, in contrast to the objective nature of spectrophotometric assessment [20]. According to Vachon et al., it has been proposed that while spectrophotometer readings may reveal a statistical disparity, these disparities may not be perceptible to the human eye from a therapeutic perspective [21]. The variations in the color shade were assessed using the Vita Lumin shade guide at three time points: baseline, day 7, and day 14. The data is gathered, organized, and analyzed using statistical methods [4].

The results of this study show a statistically significant correlation on day 7 and day 14 between the control group and members of groups I (HP), II (HPM), and III (HPMU). The baseline values of the three groups did not differ statistically. Day 7 and day 14 of Group I (HP) showed an average increase of three Vita Lumin shade tabs, while Group III (HPMU) showed an average increase of ten and three Vita Lumin shade tabs on day 7 and day 14, respectively. Group II (HPM) groups showed an average increase of six and four Vita Lumin shade tabs on day 7 and day 14.

The current study provides evidence that the use of microbubble-infused H_2_O_2_, activated by ultrasonic action, yielded superior results as an intracoronal bleaching agent for artificially stained teeth compared to plain H_2_O_2_ (35%). Microbubbles can deliver bleaching agents directly to the intracoronal area of the tooth. Further activation with ultrasound can precisely control the release of the bleaching agents from the microbubbles, ensuring they are activated only at the desired site, thereby reducing the risk of agent leakage to the cervical area. Also, microbubbles enhance the efficacy of bleaching agents, allowing the use of lower concentrations to achieve the same whitening effect. Lower concentrations reduce the risk of chemical irritation or damage to the cervical tissues, which is a potential cause of cervical resorption. Therefore, the current investigation demonstrated that the utilization of microbubbles infused with intracoronal bleaching can be considered a suitable option for the treatment of tooth discoloration.

This lab investigation cannot accurately replicate in vivo conditions in which peroxide penetration out of the root is impeded by the periodontium and physiologic or pathologic obliteration of dentinal tubules. Furthermore, periodontal inflammation can alter the extra-radicular environment, limiting the body's ability to deal with the radicals produced. Further research is required to validate these laboratory findings in clinical settings [22].

## Conclusions

The current in vitro investigation found that microbubbles containing H_2_O_2_ were more efficient and faster than plain H_2_O_2_ for intracoronal whitening of artificially stained teeth after two bleaching sessions (day 7 and day 14). Furthermore, the efficacy of bleaching was enhanced when the microbubble containing the bleaching chemical was activated using ultrasonic technology. Thus, microbubbles infused with bleaching agents can be suggested as a comparable and efficient bleaching agent for intracoronal bleaching.

## References

[1] Banomyong D (2022). Clinical considerations for non-vital tooth walking bleaching. Thai Endod J.

[2] Agrawal P, Nikhade P, Patel A, Sedani S, Bhopatkar J (2023). Management of discoloured anterior teeth with radicular cyst: a case report. Cureus.

[3] Alqahtani MQ (2014). Tooth-bleaching procedures and their controversial effects: a literature review. Saudi Dent J.

[4] Lim MY, Lum SO, Poh RS, Lee GP, Lim KC (2004). An in vitro comparison of the bleaching efficacy of 35% carbamide peroxide with established intracoronal bleaching agents. Int Endod J.

[5] Cardoso M, Martinelli CS, Carvalho CA, Borges AB, Torres CR (2013). Ultrasonic activation of internal bleaching agents. Int Endod J.

[6] AlOtaibi AlOtaibi, FL FL (2019). Adverse effects of tooth bleaching: a review. Int J Oral Care Res.

[7] Upadhyay A, Dalvi SV (2019). Microbubble formulations: Synthesis, stability, modeling and biomedical applications. Ultrasound Med Biol.

[8] Freccia WF, Peters DD (1982). A technique for staining extracted teeth: a research and teaching aid for bleaching. J Endod.

[9] D GT Jr, Gupta S, Rana KS (2023). Evaluating the effect of different intra-orifice barriers and various bleaching agents on the fracture resistance of teeth after the walking bleach procedure: an in vitro study. Cureus.

[10] Pasupathy R, Pitchaimuthu P, Subramanian S (2022). Nanobubbles: A novel targeted drug delivery system. Braz J Pharm Sci.

[11] Ganesh R, Aruna S, Joyson M (2013). Comparison of the bleaching efficacy of three different agents used for intracoronal bleaching of discolored primary teeth: an in vitro study. J Indian Soc Pedod Prev Dent.

[12] Martin-Biedma B, Gonzalez-Gonzalez T, Lopes M, Lopes L, Vilar R, Bahillo J, Varela-Patiño P (2010). Colorimeter and scanning electron microscopy analysis of teeth submitted to internal bleaching. J Endod.

[13] Abbott P, Heah SY (2009). Internal bleaching of teeth: an analysis of 255 teeth. Aust Dent J.

[14] Fasanaro TS (1992). Bleaching teeth: history, chemicals, and methods used for common tooth discolorations. J Esthet Dent.

[15] Pearson HH (1951). Successful bleaching without secondary discolouration. J Can Dent Assoc (Tor).

[16] Amer M (2023). Intracoronal tooth bleaching - a review and treatment guidelines. Aust Dent J.

[17] Friedman S (1997). Internal bleaching: long-term outcomes and complications. J Am Dent Assoc.

[18] Camps J, de Franceschi H, Idir F, Roland C, About I (2007). Time-course diffusion of hydrogen peroxide through human dentin: clinical significance for young tooth internal bleaching. J Endod.

[19] Kwon SR, Wertz PW (2015). Review of the mechanism of tooth whitening. J Esthet Restor Dent.

[20] Tran L, Orth R, Parashos P (2017). Depletion rate of hydrogen peroxide from sodium perborate bleaching agent. J Endod.

[21] Vachon C, Vanek P, Friedman S (1998). Internal bleaching with 10% carbamide peroxide in vitro. Pract Periodontics Aesthet Dent.

[22] Zoya A, Tewari RK, Mishra SK (2019). Sodium percarbonate as a novel intracoronal bleaching agent: assessment of the associated risk of cervical root resorption. Int Endod J.

